# Supplementary material for: Stromal Senescence following Treatment with the CDK4/6 Inhibitor Palbociclib Alters the Lung Metastatic Niche and Increases Metastasis of Drug-Resistant Mammary Cancer Cells
Source: Cancers (Basel). 2023 Mar 22;15(6):1908. doi: 10.3390/cancers15061908 (PMC10046966; doi:10.3390/cancers15061908)
Supplement: Supplementary file 1 [file cancers-15-01908-s001.zip › Supplemental figures.pdf]

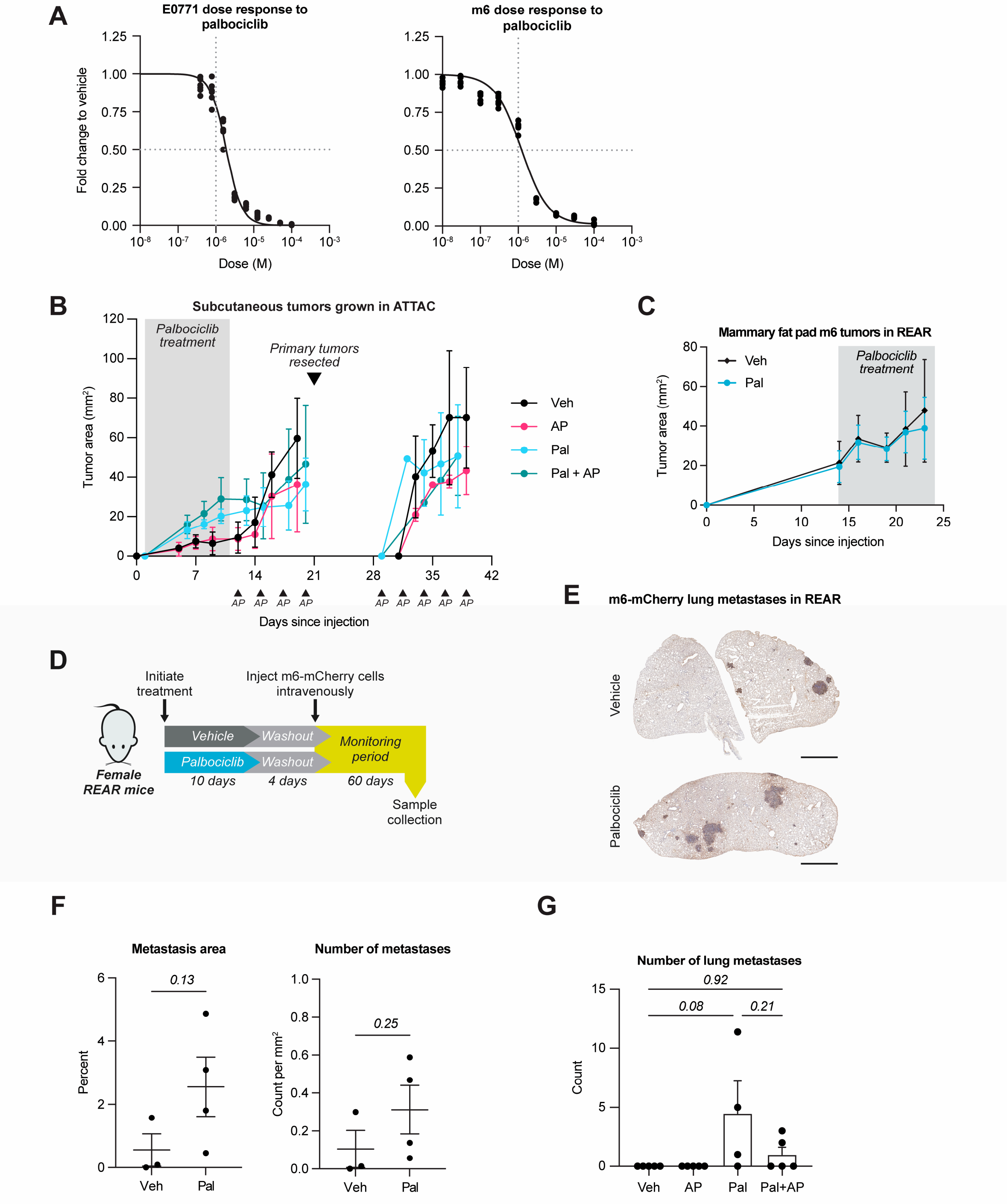

Supplemental Figure S1: Comparison of m6 and E0771 cell line sensitivity to palbociclib *in vitro* and *in vivo*. (**A**) Dose-response growth curves by crystal violet stain of E0771 and m6 murine cell lines to palbociclib. E0771 IC_{50} = 1.889 μM; m6 IC_{50} = 1.220 μM. Sensitivity threshold of 1 μM dose indicated by dotted line. Fold change in absorbance compared to vehicle plotted. (**B**) Assessment of *in vivo* sensitivity of E0771 to palbociclib with or without AP20187 (AP). 10^6 E0771 cells were implanted subcutaneously in the right and left flanks of female ATTAC mice. Mice were treated with vehicle (Veh), AP20187 (AP), palbociclib (Pal), or palbociclib and AP combination (Pal + AP). One day after injection, treatment with 150 mg/kg daily palbociclib or vehicle control was initiated for 10 days (10 total doses). One day after completion of the palbociclib treatment, treatment with AP 2 mg/kg or vehicle control was initiated every three days for 10 days (four doses). Tumors were resected 21 days after implantation. Mice were maintained for 7 days and then treated with five additional doses of AP 2 mg/kg or vehicle every three days before collection. Tumor area (L × W) was measured at regular intervals

throughout; mean ± SD graphed. (**C**) Assessment of *in vivo* sensitivity of m6 to palbociclib. 10^6 m6 cells in 50% Matrigel/PBS mix were implanted percutaneously into the right and left inguinal mammary glands of female REAR mice. Fourteen days after injection, treatment with 150 mg/kg daily palbociclib or vehicle control was initiated for 10 days (10 total doses). Tumor area (L x W) calculated by measurements over the skin and measured at regular intervals up to 24 days after injection; mean ± SD graphed. (**D**) Experimental schematic for (**E**,**F**): REAR mice received 10 days of treatment with 150 mg/kg palbociclib daily by oral gavage (10 total doses) followed by a 96 h drug washout period and injection of 10^6 m6-mCherry cells intravenously in the tail vein. After injection, mice were monitored for 60 days and then lung tissue was collected. (**E**) Representative lung sections from REAR mice bearing m6-mCherry metastases with immunohistochemistry stain for SV40 T antigen. Scale bars = 2 mm. (**F**) REAR lung area occupied by m6-mCherry metastasis quantified as cross-sectional metastasis area as a percentage of lung area compared by unpaired *t*-test. (**G**) Number of metastatic foci in lungs of ATTAC mice from experiment described in (B). Compared by one-way ANOVA with multiple comparisons test with Tukey's multiple comparisons test. (**F**,**G**) Mean ± standard error graphed; *p*-values indicated.

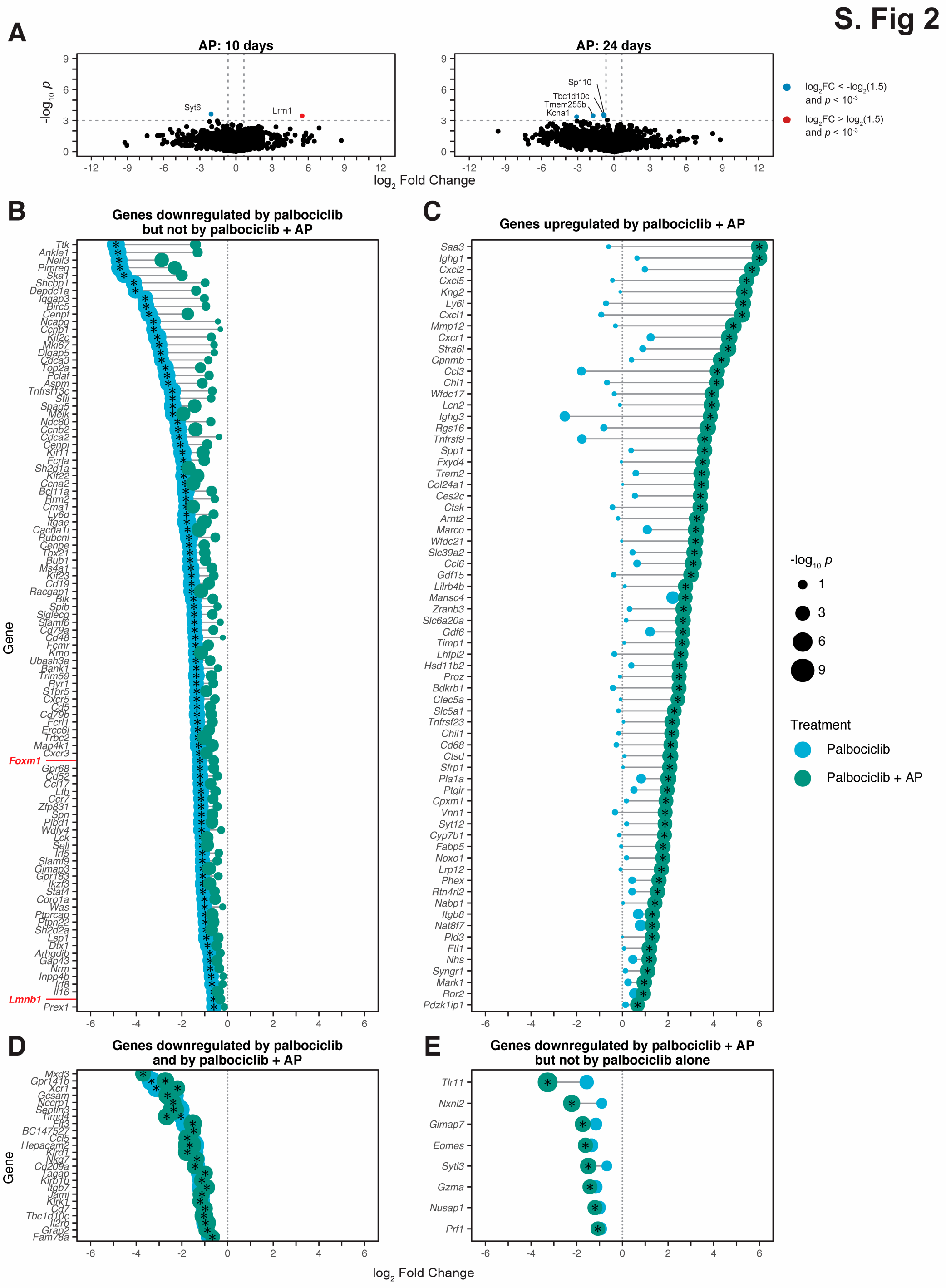

Supplemental Figure S2. Differential gene expression at 10 days in ATTAC mouse lungs treated with 10 days of palbociclib or palbociclib + AP20187. (**A**) Differentially expressed genes (DEGs) from n = 3 mice treated with 10 days of AP 2 mg/kg every three days (four doses) or from n = 3 mice treated with 24 days of AP 2 mg/kg every three days (nine total doses) versus mice that received DMSO vehicle every three days for 10 days (n = 3) or 24 days (n = 3). Color indicates genes that surpassed statistical significance threshold of absolute value of $\log_2$ fold change > $\log_2(1.5)$ and $p < 10^{-3}$. (**B**) Genes were significantly downregulated by 10 days of palbociclib treatment but not by 10 days of palbociclib + AP, including *Foxm1* and *Lmnb1*, which were lost during senescence. (**C**) Genes significantly upregulated by 10 days of palbociclib + AP. (**D**) Genes significantly downregulated by 10 days of palbociclib treatment and by 10 days of palbociclib + AP. (**E**) Genes significantly downregulated by palbociclib + AP but not by palbociclib alone. (**B**–**E**) Color indicates treatment; size indicates significance; * = differential gene expression surpassed statistical significance threshold of absolute value of $\log_2$ fold change > $\log_2(1.5)$ and $p < 10^{-3}$.

S. Fig 3

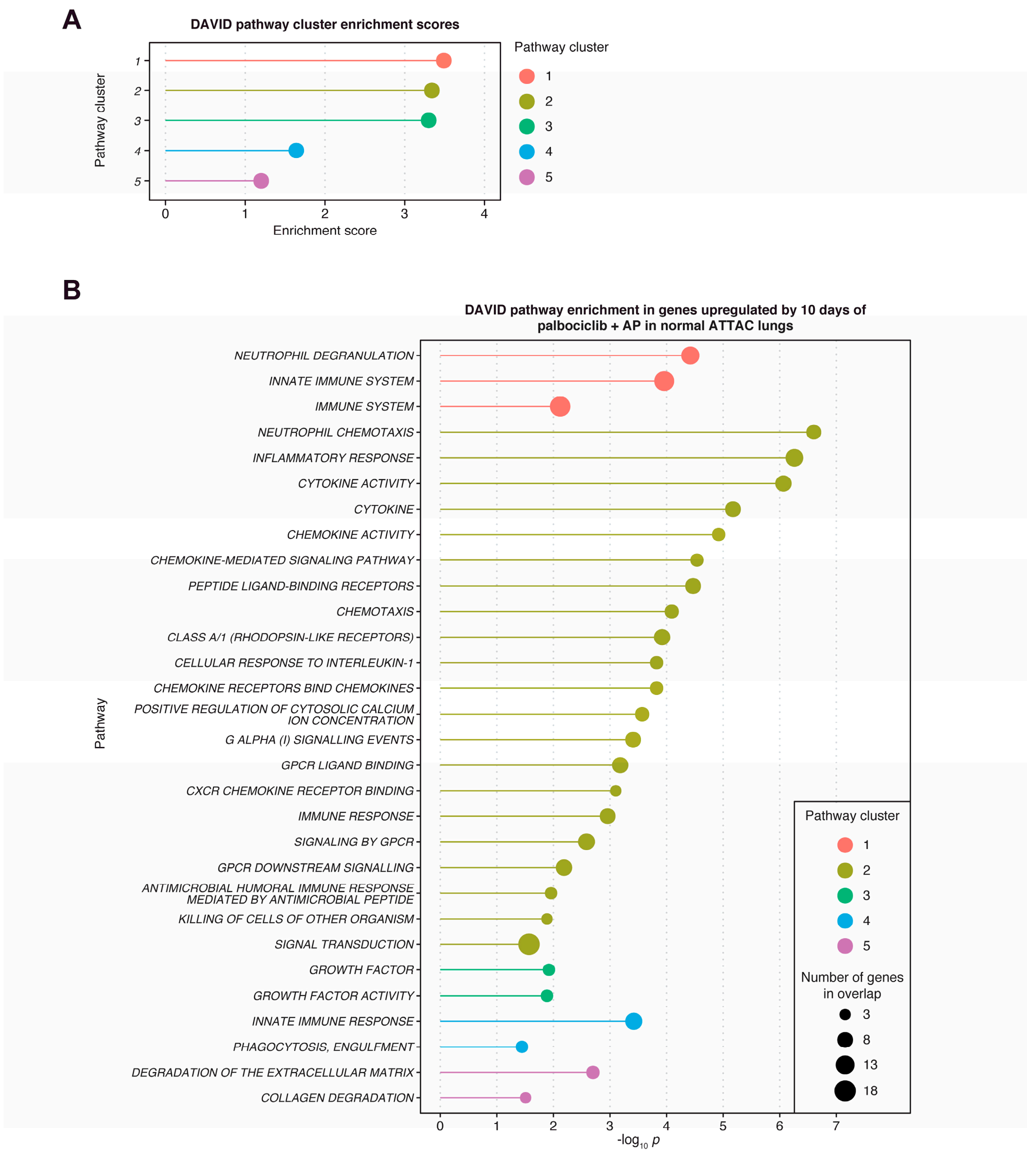

Supplemental Figure S3. Gene ontology enrichment in genes upregulated by 10 days of palbociclib + AP20187 (AP) in normal lungs. (**A**) Annotation cluster enrichment scores from gene ontology analysis using DAVID for the sixty-eight genes upregulated by 10 days of palbociclib + AP treatment with $\log_2$(fold change) > 1.5 and $p < 10^{-3}$. Five annotation clusters with DAVID-generated enrichment scores greater than 1.0 were generated using the default settings. (**B**) Enriched annotation terms with p-value of overlap < 0.05 that contributed to each annotation cluster from the analysis described in (**A**). Color indicates the membership of each term to an annotation cluster; size indicates the number of genes from the query set that overlap with genes associated with each annotation term.

S. Fig 4

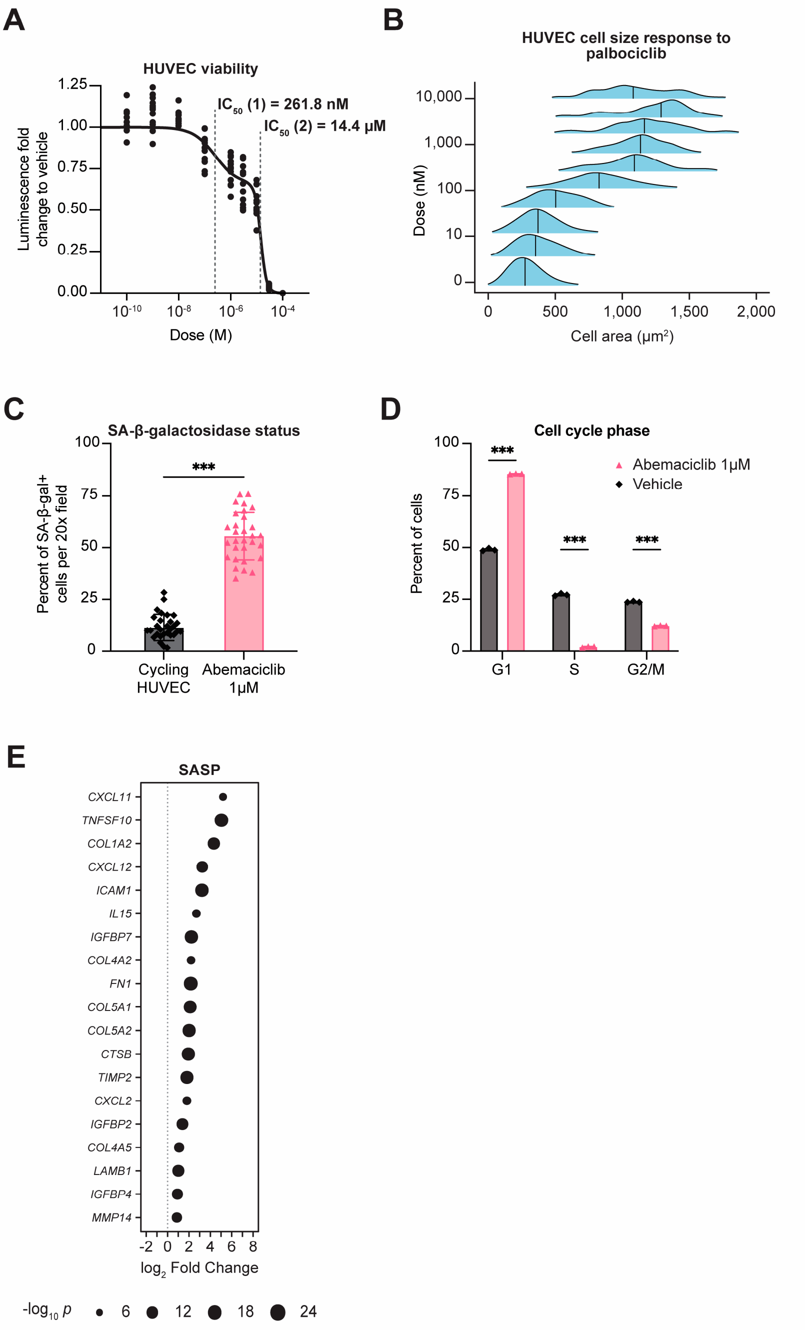

Supplemental Figure S4. HUVEC demonstrate hallmark features of senescence in response to both palbociclib and abemaciclib *in vitro*. (**A**) Viability of HUVEC treated with increasing doses of palbociclib. A biphasic sigmoid curve was fitted to luminescence values from CellTiter-Glo assay normalized to vehicle; $n = 6$ independent replicates per dose. (**B**) Area occupied per cell of HUVEC treated with palbociclib *in vitro*. Quantified from bright-field microscopy. Graphical representation reflects relative distribution of values with the median cell area indicated with a line; $n > 250$ cells counted per dose. (**C**) Senescence-associated β-galactosidase expression status of HUVEC treated with 1 µM abemaciclib versus cycling HUVEC. Thirty non-overlapping 20x fields captured per dose compared by unpaired *t*-test. (**D**) Cell cycle status of HUVEC treated with abemaciclib for 72 h compared to cycling HUVEC, quantified by propidium iodide stain and flow cytometry. Three independent replicates per treatment condition compared by two-way ANOVA with Tukey's multiple comparisons test. (**C**,**D**) Cycling HUVEC collected 24 h after cryorecovery and one subsequent passage. All bars indicate mean ± standard error; *** = $p < 0.001$. (**E**) Differentially expressed genes in palbociclib-treated HUVEC related to the senescence-associated secretory phenotype [14]. All genes filtered for absolute value of $\log_2$ fold change > $\log_2(1.5)$ and $p < 10^{-6}$ or were excluded. Size indicates significance.
